# Evaluating the cytotoxicity of innate immune effector cells using the GrB ELISPOT assay

**DOI:** 10.1186/1479-5876-2-31

**Published:** 2004-09-20

**Authors:** Kimberly A Shafer-Weaver, Thomas Sayers, Douglas B Kuhns, Susan L Strobl, Mark W Burkett, Michael Baseler, Anatoli Malyguine

**Affiliations:** 1 Laboratory of Cell-Mediated Immunity, SAIC-Frederick, Inc., National Cancer Institute at Frederick, Frederick, MD USA; 2 Clinical Services Program, SAIC-Frederick, Inc., National Cancer Institute at Frederick, Frederick, MD USA; 3 Laboratory of Experimental Immunology, Intramural Research Support Program, SAIC-Frederick, Inc., National Cancer Institute at Frederick, Frederick, MD USA; 4 Neutrophil Monitoring Laboratory, SAIC-Frederick, Inc., National Cancer Institute at Frederick, Frederick, MD USA

## Abstract

**Background:**

This study assessed the Granzyme B (GrB) ELISPOT as a viable alternative to the ^51^Cr-release assay for measuring cytotoxic activity of innate immune effector cells. We strategically selected the GrB ELISPOT assay because GrB is a hallmark effector molecule of cell-mediated destruction of target cells.

**Methods:**

We optimized the GrB ELISPOT assay using the human-derived TALL-104 cytotoxic cell line as effectors against K562 target cells. Titration studies were performed to assess whether the ELISPOT assay could accurately enumerate the number of GrB-secreting effector cells. TALL-104 were treated with various secretion inhibitors and utilized in the GrB ELISPOT to determine if GrB measured in the ELISPOT was due to degranulation of effector cells. Additionally, CD107a expression on effector cells after effector-target interaction was utilized to further confirm the mechanism of GrB release by TALL-104 and lymphokine-activated killer (LAK) cells. Direct comparisons between the GrB ELISPOT, the IFN-γ ELISPOT and the standard ^51^Cr-release assays were made using human LAK cells.

**Results:**

Titration studies demonstrated a strong correlation between the number of TALL-104 and LAK effector cells and the number of GrB spots per well. GrB secretion was detectable within 10 min of effector-target contact with optimal secretion observed at 3–4 h; in contrast, optimal IFN-γ secretion was not observed until 24 h. The protein secretion inhibitor, brefeldin A, did not inhibit the release of GrB but did abrogate IFN-γ production by TALL-104 cells. GrB secretion was abrogated by BAPTA-AM (1,2-bis-(2-aminophenoxy)ethane-N,N,N', N'-tetraacetic acid tetra(acetoxymethyl) ester), which sequesters intracellular Ca^2+^, thereby preventing degranulation. The number of effector cells expressing the degranulation associated glycoprotein CD107a increased after interaction with target cells and correlated with the stimulated release of GrB measured in the ELISPOT assay.

**Conclusions:**

Because of its high sensitivity and ability to estimate cytotoxic effector cell frequency, the GrB ELISPOT assay is a viable alternative to the ^51^Cr-release assay to measure MHC non-restricted cytotoxic activity of innate immune cells. Compared to the IFN-γ ELISPOT assay, the GrB ELISPOT may be a more direct measure of cytotoxic cell activity. Because GrB is one of the primary effector molecules in natural killer (NK) cell-mediated killing, detection and enumeration of GrB secreting effector cells can provide valuable insight with regards to innate immunological responses.

## Background

Cytotoxic T-lymphocytes (CTL) and natural killer (NK) cells play an important role in host defense against intracellular pathogens and tumor cells. CTL recognize target cells through processed antigenic peptides presented via MHC. In contrast, NK cells mediate lysis of numerous cellular targets without classical MHC restriction. NK cells appear to use a variety of different, non-rearranging receptors to initiate cytoxicity and cytokine production [1]. Although CTL and NK differ in the receptors they use to recognize target cells, they both utilize the granule exocytosis and the Fas ligand (FasL)-mediated pathways to eliminate altered-self targets [2-6]. The granule-mediated pathway is the dominant pathway in CTL and NK [5]. CTL and NK cell granules contain a number of proteins, including perforin and granzymes, with GrB being the most abundant granzyme present [7,8]. Upon recognition and conjunction of the effector cell with the target, preformed granules containing GrB polarize in cytolytic lymphocytes at the point of contact and are secreted into the intercellular space formed between the effector and target cell [9-14]. The secretion of GrB occurs quite rapidly, is Ca^2+^-dependent, and mediates the lethal hit that kills virus-infected and tumor cells [2,7,8,10,15-19].

Cell-mediated cytotoxicity has conventionally been measured using the standard ^51^Cr-release assay [20]. Recently, the use of the IFN-γ ELISPOT assay as a surrogate measure for CTL and NK responses has gained increased application. However, the IFN-γ ELISPOT assay may not be an accurate measure of cytotoxic lymphocytes as non-cytotoxic cells can secrete IFN-γ. Since GrB is exclusively present in the granules of CTL and NK cells and is a key mediator of the granule exocytosis-mediated cytolytic pathway [21-23], it is an excellent candidate marker for immunological monitoring of innate immunity by the ELISPOT method.

The ELISPOT method has been successfully applied to measure GrB secretion by GrB-transfected CHO cells and for assessing antigen specific T-cell cytotoxic activity [24,25]. In this study, we utilized human NK-like and lymphokine-activated killer (LAK) effector cells to assess whether the GrB ELISPOT assay could accurately measure the MHC non-restricted cytolysis that occurs upon recognition of appropriate target cell ligands by activating receptors on these effector cells. Additionally, we evaluated whether the ELISPOT assay measured GrB release due to degranulation of stimulated effector cells. The GrB ELISPOT assay was strategically compared to the IFN-γ ELISPOT and the standard ^51^Cr-release assays to determine if the GrB ELISPOT assay is a viable or better alternative to measure innate immunity.

## Methods

### Target cell lines

K562 cells (Human myelogenous leukemia cell line, ATCC, Manassas, VA) were cultured at 37°C, 5% CO_2 _in tissue culture medium (TCM) consisting of RPMI 1640 (BioWhittaker, Walkersville, MD) supplemented with 10% fetal bovine serum (FBS; Hyclone, Logan, UT), 1 mM non-essential amino acids, 2 mM glutamine, 100 U/ml Penicillin, 100 μg/ml Streptomycin, 20 mM HEPES and 1 mM sodium pyruvate (Gibco BRL Life Technologies, Grand Island, NY).

### TALL-104 effector cells

The TALL-104 cell line is a T-cell derived line that is highly cytotoxic for NK-sensitive targets and when grown in the presence of IL-2, can be stimulated to secrete IFN-γ and GrB [26,27]. The cell line was cultured in Iscove's modified Dulbecco's medium (BioWhittaker) supplemented with 20% FBS (Hyclone), 4 mM glutamine, 100 U/ml Penicillin and 100 μg/ml Streptomycin (Gibco BRL Life Technologies) at 8% to 10% CO_2_. Recombinant human IL-2 (100 U/ml; Hoffmann-LaRoche, Nutley, NJ) was added every 2–3 days to ensure optimal growth and maintenance of cytotoxic activity.

### Generation of human lymphokine-activated killer (LAK) cells

Peripheral blood mononuclear cells (PBMC) were isolated from venous blood of normal human volunteers by buoyant density centrifugation over Ficoll-Paque (Pharmacia, Piscataway, NJ). Aliquots of effector cells were cryopreserved in the vapor phase of liquid N_2 _for future use in functional testing. The PBMC were thawed and resuspended at 2 × 10^6 ^cells/ml in 20 ml of RPMI 1640 (BioWhittaker) containing 10% human AB serum (Mediatech, Herndon, VA), 1 mM non-essential amino acids, 2 mM glutamine, 1 mM pyruvate, 20 mM HEPES, 100 U/ml Penicillin and 100 μg/ml Streptomycin (Gibco BRL Life Technologies). Cell suspensions were stimulated with 100 U/ml of IL-2 (Hoffmann-LaRoche) on day 0 and cultured for 5 to 6 days at 37°C, 5% CO_2_. LAK cultures consisted of 30.0 ± 2.1 % NK cells (CD3^-^,CD16/CD56^+^) and 26.9 ± 4.9 CD8^+ ^T-cells as determined by flow cytometric analysis.

### Secretion Inhibitors

TALL-104 cells were treated with inhibitors of cellular secretion prior to use in the GrB and IFN-γ ELISPOT assays. Inhibitors used included 1,2-bis-(2-aminophenoxy)ethane-N,N,N', N'-tetraacetic acid tetra(acetoxymethyl) ester (BAPTA-AM, Molecular Probes, Eugene, OR), a cell permeant chelator of intracellular Ca^2+^, and γ,4-dihydroxy-2-[6-hydroxy-1-hep-tenyl]-4-cyclopentanecrotonic acid λ-lactone (brefeldin A; Sigma, St. Louis, MO) which blocks protein secretion. TALL-104 cells were resuspended at 2.5 × 10^5 ^cells/ml and 2.5 × 10^4 ^cells/ml in PBS without divalent cations for BAPTA-AM and brefeldin A treatment, respectively. Cells were pretreated with the indicated concentrations of BAPTA-AM for 45 min or with 5 μg/ml brefeldin A for 1 h at 37°C, washed twice, assessed for viability by trypan blue exclusion, and resuspended at 2.5 × 10^4 ^cells/ml in assay media.

### ^51^Cr-release assay

Cytotoxicity of TALL-104 and LAK cells was assessed using the standard ^51^Cr-release assay. Briefly, one million target cells were labeled at 37°C for 1 h with 100 μCi Na_2_^51^CrO_4 _(New England Nuclear, Boston, MA). Target cells were washed and resuspended in TCM at 5 × 10^4 ^cells/ml. Five thousand target cells per well (100 μl) were added to a 96 well plate (Costar, Cambridge, MA) following the appropriate number of effector cells (100 μl/well). The defined effector:target (E:T) ratios were plated in triplicate. Cytotoxicity assays were performed at 37°C for 4 h. Percent specific lysis was calculated using the following equation:

(ER - SR)/(MR - SR) × 100,

where ER = experimental release, SR = spontaneous release and MR = maximum release.

### Granzyme B ELISPOT assay

Granzyme B secretion was measured using the GrB ELISPOT assay as previously described [25]. Briefly, MultiScreen-IP plates (PVDF membrane, Millipore, Bedford, MA) were coated overnight at 4°C with 100 μl/well of anti-human GrB antibody (7.5 μg/ml in PBS, clone GB-10, PeliCluster, Cell Sciences, Norwood MA). Effector cells (100 μl/well) were added to triplicate wells at specified concentrations followed by 5 × 10^4 ^target cells per well (100 μl). After the specified effector-target cell incubation, the plates were washed and 100 μl/well of biotinylated anti-human GrB detecting antibody (0.25 μg/ml in PBS/1% BSA/0.05% Tween 20, clone GB-11, PeliCluster, Cell Sciences) was added. Plates were incubated for 3 h and 50 μl of Streptavidin-Alkaline Phosphatase (1:1500 in PBS/1% BSA, Gibco BRL Life Technologies) was added for 1 h. Spots were visualized with 100 μl/well of BCIP-NBT phosphatase substrate (KPL, Gaithersburg, MD) and subjected to automated evaluation using the ImmunoSpot Imaging Analyzer system (Cellular Technology Ltd, Cleveland, OH).

### IFN-γ ELISPOT assay

For assessing IFN-γ secretion, MultiScreen-IP plates (PVDF membranes, Millipore) were coated overnight at room temperature with 50 μl/well of anti-human IFN-γ antibody (20 μg/ml in PBS, Biosource, International, Camarillo CA) as previously described [25]. After effector and target cells were incubated at 37°C, the plates were washed and 50 μl/well of biotinylated anti-human IFN-γ antibody (1.3 μg/ml in PBS/1% BSA/0.05% Tween 20, BD PharMingen, San Jose, CA) was added. Plates were incubated with biotinylated antibody, washed and 50 μl of Streptavidin-Alkaline Phosphatase (1:1500 in PBS/1% BSA, Gibco BRL Life Technologies) was added. Spots were visualized and enumerated as described above.

### CD107a mobilization assay

Degranulation of TALL-104 and LAK cells in response to target cell recognition was assessed by monitoring surface antigen expression of CD107a (lysosomal-associated membrane protein-1), a surface antigen transiently present on the cell surface after release of cytolytic granules. Expression of CD107a has been used as a marker to measure degranulation by flow cytometry [28,29].

To distinguish target cells from effector cells, K562 cells were first labeled with PKH67 dye (Sigma) according to the manufacturer's instructions and washed extensively prior to use in the assay. Effector cells (2 × 10^5^) were resuspended in 400 μl of phenol red-free TCM in polystyrene tubes (Falcon, Franklin Lakes, NJ). PKH67-labeled K562 target cells (1 × 10^5 ^cells in 50 μl) were then added to appropriate tubes, the tubes were spun for 30 sec at 500 rpm and incubated for the specified periods in a 37°C water bath. Reactions were quenched with cold PBS and then 20 μl of CD107a-PE-Cy5 (PharMingen) was added. Mouse IgG_1_-biotin (Beckman-Coulter, Miami, FL) and streptavidin-PE-Cy5 (Caltag, Burlingame, CA) were utilized for controls. Tubes were incubated with antibody for 20 min at room temperature, washed with buffer and analyzed using a FACScan instrument (Becton-Dickinson Immunocytometry Systems, San Jose, CA) equipped with a 15 mW argon-ion laser. The percent of effector cells expressing CD107a was determined by gating the PKH67 negative (effector cells) population. Within the PKH67 negative gate, CD107a expression was determined from histograms based on forward and side scatter analysis. Effector cells run in the absence of target cells were evaluated for baseline expression of CD107a. The percentage of effector cells induced by target cells to express CD107a was calculated by subtracting background (spontaneous) expression from experimental samples.

### Statistical analysis

Statistical analysis was performed using Student's *t *test and Pearson correlation coefficient (R^2^).

## Results

### GrB release by stimulated TALL-104 cells

**Figure 1:**
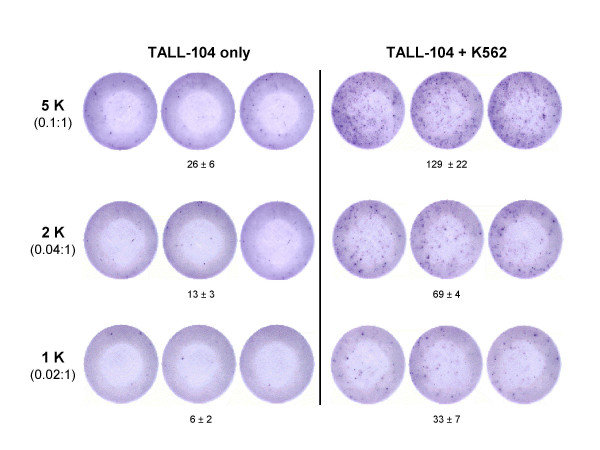
**Granzyme B release by stimulated TALL-104 cells measured in the ELISPOT assay. **TALL-104 cells (1 × 10^3^, 2 × 10^3 ^or 5 × 10^3 ^cells per well) were incubated with K562 targets (5 × 10^4 ^cells per well) for 4 h at 37°C in the Granzyme B ELISPOT assay (effector:target cell ratios are in brackets). Data are presented as average number of spots per well ± SD and are representative of 3 experiments with similar results. A significant correlation between the number of spots per well and effector cell number was observed (R^2 ^= 0.98).

GrB ELISPOT assays were performed using a constant number of K562 targets and decreasing numbers of TALL-104 effector cells per well. As the number of TALL-104 cells decreased, the resulting number of GrB spots decreased linearly. Limited GrB spots were detected in wells containing TALL-104 alone demonstrating that the ELISPOT assay measured primarily stimulated release of GrB (Fig. 1). K562 alone did not secrete GrB (data not shown). The correlation between the number of spots per well and the number of TALL-104 was highly significant with a Pearson correlation coefficient of R^2 ^= 0.98. Therefore, the GrB ELISPOT assay is capable of measuring the frequency of effector cells stimulated to secrete GrB.

### Correlation of GrB secretion and cytotoxic activity

In tandem with the GrB ELISPOT assay, TALL-104 cells were also utilized as effectors against K562 targets in the standard ^51^Cr-release assay to assess lytic activity. Significant specific lysis was observed at effector:target ratios from 10:1 to 1:1 (Table 1). In contrast to the ELISPOT assay, that can measure the frequency of cytotoxic cells at effector:target ratios as low as 0.02:1, specific lysis was not significant in the ^51^Cr-release assay at cell ratios below 1:1 (Table 1). However, when optimal ratios were used in each individual assay, similar trends between the number of GrB spots per well in the ELISPOT and specific lysis in the ^51^Cr-release assay were observed. These data demonstrate that the release of GrB is indicative of cytolytic activity.

**Table 1:** Comparison of the GrB ELISPOT and ^51^Cr-release assays for measuring TALL-104 cell activity.

Effector:Target Ratio	^51^Cr-Release Assay^a ^(% Cytotoxicity ± SE)	GrB ELISPOT Assay^b ^(Spots/well ± SE)
10:1	65 ± 4	TNTC^c^
5:1	57 ± 10	TNTC
2.5:1	47 ± 11	TNTC
1:1	20 ± 5	TNTC
0.5:1	9 ± 2	TNTC
0.2:1	2 ± 1	316 ± 50
0.1:1	2 ± 1	144 ± 13
0.05:1	1 ± 1	80 ± 6
0.02:1	0 ± 1	33 ± 4

^a ^The ^51^Cr-release assay was performed with the effector:target cell ratio as specified for 4 h at 37°C. Data presented as average cytotoxicity ± SE (n = 3).

^b^The granzyme B ELISPOT assay was performed with K562 cells at a constant number of 5 × 10^4 ^cells per well and effector:target cell ratio as specified for 4 h at 37°C. Data presented as average spots/well ± SE (n = 3).

^c^TNTC= Too numerous to count accurately.

### Secretion kinetics of GrB and IFN-γ

**Figure 2:**
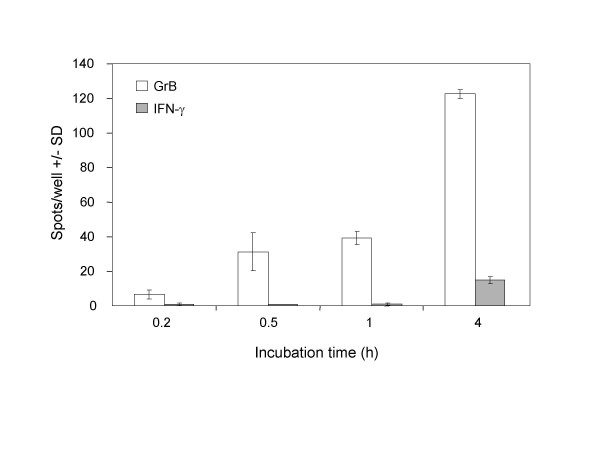
**Kinetics of Granzyme B and IFN-γ secretion by TALL-104 cells in the ELISPOT assays. **TALL-104 cells (2.5 × 10^3 ^cells per well) were incubated with K562 cells (5 × 10^4 ^cells per well) in the Granzyme B and IFN-γ ELISPOT assays for 0.2, 0.5, 1, or 4 h at 37°C. Data are presented as average spots per well ± SD. Results are corrected for background and are representative of 3 experiments with similar results.

To measure the secretion kinetics of GrB compared to IFN-γ, TALL-104 cells were incubated with K562 target cells for the indicated times in the ELISPOT assays. Secretion of GrB was compared to IFN-γ because IFN-γ is currently the standard utilized to measure the frequency of cytotoxic cells via the ELISPOT method. GrB secretion was observed within 10 min of effector-target cell interaction with optimal secretion around 3–4 h (Fig. 2). This observation is in contrast to maximum IFN-γ production, which was observed at 24 h of effector-target interaction (data not shown). These data are consistent with rapid effector cell degranulation (within minutes) upon contact with target cells [18,19].

### Mechanism of GrB secretion

Effects of secretion inhibitors on the release of GrB and IFN-γ in the ELISPOT assays as well as expression of CD107a on effector cells were evaluated to determine the possible mechanism of GrB release measured in the ELISPOT assay. TALL-104 cells were treated with inhibitors of cellular secretion and assessed for their ability to release GrB and IFN-γ in the ELISPOT assays. BAPTA-AM chelates intracellular Ca^2+ ^and was utilized to block degranulation. Brefeldin A, which blocks protein secretion, was utilized to prevent secretion of newly synthesized proteins.

**Figure 3:**
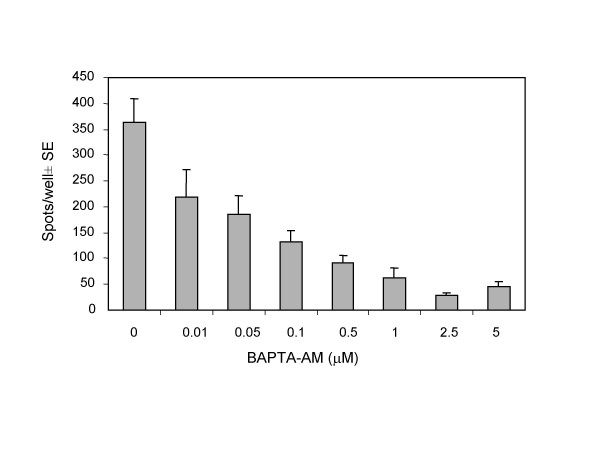
**Effect of BAPTA-AM on Granzyme B secretion by TALL-104 cells. **TALL-104 cells were pretreated with the indicated concentrations of BAPTA-AM for 45 min at 37°C. TALL-104 cells (2.5 × 10^3 ^cells per well) were incubated with K562 (5.0 × 10^4 ^cells per well) for 4 h in the Granzyme B ELISPOT assay. Data are presented as spots per well ± SE (n = 3). Results are corrected for background.

**Figure 4:**
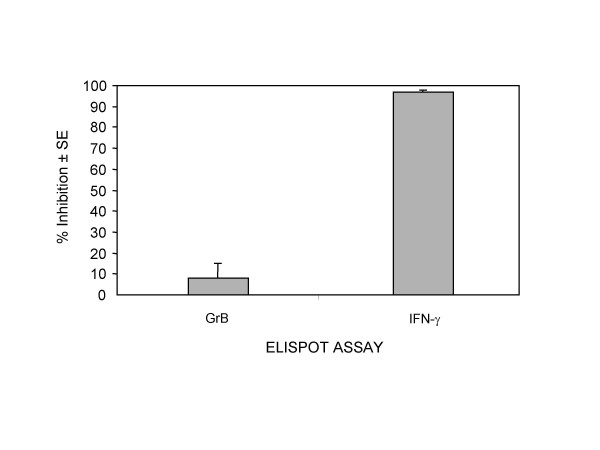
**Effect of Brefeldin A on Granzyme B and IFN-γ secretion in the ELISPOT assays. **TALL-104 cells were preincubated with brefeldin A (5 μg/ml; 1 h at 37°C) prior to use in the ELISPOT assays. TALL-104 cells (2.5 × 10^3 ^cells per well) were incubated with K562 (5 × 10^4 ^cells per well) for 4 h in the Granzyme B and 24 h in the IFN-γ ELISPOT assays at 37°C. Data are presented as percent inhibition ± SE (n = 3). Results are corrected for background.

**Figure 5:**
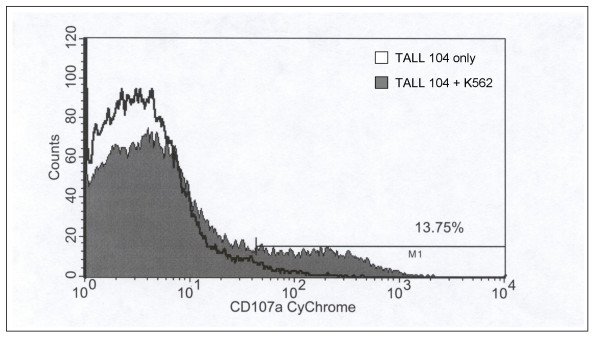
**Stimulated TALL-104 cells express the degranulation marker CD107a. **TALL-104 (2 × 10^5 ^cells) were incubated with K562 (1 × 10^5 ^cells) and surface expression of CD107a was determined by flow cytometric analysis. K562 were prelabeled with PKH67 dye to differentiate effector cells from targets. The histograms represent the PE-Cy5 CD107a positive cells determined by forward versus side scatter of the gated effector cells. TALL-104 cells alone are represented by the open histogram and TALL-104 cells incubated with K562 for 1 h are represented by the shaded histogram. The data is representative of 3 experiments with similar results.

Loading TALL-104 cells with BAPTA-AM resulted in a dose-dependent inhibition of GrB secretion (Fig. 3). Inhibition of GrB was not attributed to alterations in cell viability as TALL-104 cells loaded with as high as 100 μM of BAPTA-AM remained viable as assessed by trypan blue exclusion (data not shown). Brefeldin A treatment did not alter the secretion of GrB when TALL-104 cells were stimulated with K562 targets but did significantly (*p *< 0.05) inhibit IFN-γ secretion (Fig. 4). Additionally, increased expression of the degranulation marker, CD107a, on TALL-104 cells was observed when TALL-104 cells were incubated with K562 target cells. Compared to TALL-104 cells alone, the addition of K562 targets induced 13.75% of TALL-104 cells to express CD107a (Fig. 5). Taken together, these data are consistent with synthesis of IFN-γ *de novo *while GrB is released from preformed granules upon effector-target interaction.

### Application of the GrB ELISPOT assay to assess innate immunity

**Figure 6:**
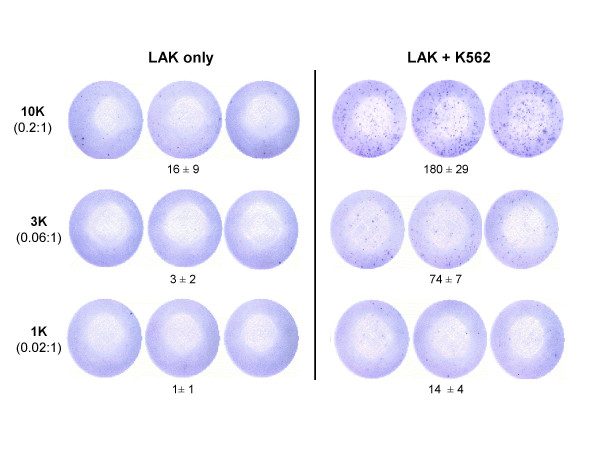
**Granzyme B release by stimulated LAK cells measured in the ELISPOT assay. **Human peripheral blood mononuclear cells (PBMC) were cultured with 100 U/ml of IL-2 for 5–6 days to induce LAK cells. LAK cells (1 × 10^3^, 3 × 10^3 ^or 1 × 10^4 ^cells per well) were incubated with K562 (5 × 10^4 ^cells per well) for 4 h at 37°C. Effector:target cell ratios are in brackets. Data are presented as average number of spots per well ± SD and are representative of 5 experiments with similar results. A significant correlation between the number of spots per well and effector cell number was observed (R^2 ^= 0.94).

To address the potential clinical relevance of the GrB ELISPOT assay, LAK cells derived from PBMC, rather than the TALL-104 cell line, were utilized as effector cells. As shown in Figure 6, the number of GrB spots per well correlated with the number of LAK cells added, results comparable to those obtained with TALL-104 cells. Only wells containing both LAK and K562 target cells contained a substantial number of spots (Fig. 6).

**Figure 7:**
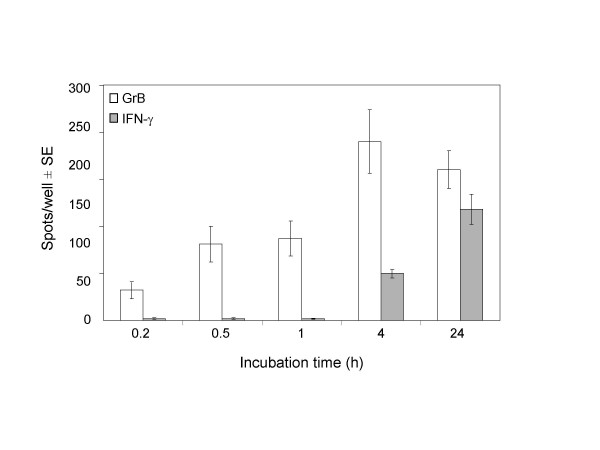
**Kinetics of Granzyme B and IFN-γ secretion by LAK cells in the ELISPOT assays. **Human peripheral blood mononuclear cells (PBMC) were cultured with 100 U/ml of IL-2 for 5–6 days to induce LAK cells. LAK cells (5 × 10^3 ^cells per well) were incubated with K562 cells (5 × 10^4 ^cells per well) in the Granzyme B and IFN-γ ELISPOT assays for 0.2, 0.5, 1, 4 or 24 h at 37°C. Data are presented as average spots per well ± SE (n = 6). Results are corrected for background.

**Figure 8:**
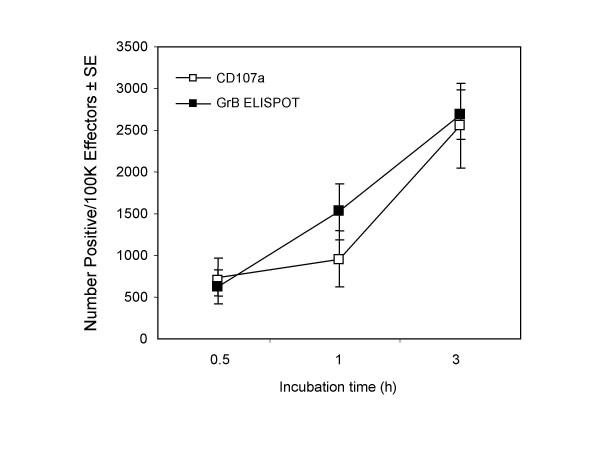
**Correlation between CD107a expression measured in the flow cytometric assay and Granzyme B release measured in the ELISPOT assay. **Human peripheral blood mononuclear cells (PBMC) were cultured with 100 U/ml of IL-2 for 5–6 days to induce LAK cells. LAK cells (5 × 10^3 ^cells per well) were incubated with K562 cells (5 × 10^4 ^cells per well) in the Granzyme B ELISPOT and 2 × 10^5 ^LAK cells were incubated with 1 × 10^5 ^K562 in the CD107a mobilization assay for 0.5, 1 and 3 h. The ELISPOT data are presented as spots per 1.0 × 10^5 ^effectors ± SE (n = 9) and the CD107a data as the number of positive cells per 1.0 × 10^5 ^effectors ± SE (n = 9). All results are corrected for background. A significant correlation between the number of GrB spots and CD107a positive effector cells was observed (R^2 ^= 0.89).

The secretion kinetics of GrB and IFN-γ by LAK cells were similar to that observed for TALL-104 cells. GrB secretion was seen within 10 min of effector-target cell interaction with optimal secretion around 3 h and maximal IFN-γ production was observed at 24 h of effector-target interaction (Fig. 7). Similarly, stimulated secretion of GrB by LAK cells was associated with increased surface expression of the degranulation marker, CD107a. The correlation between the number of GrB spots and the number of cells expressing CD107a per 100,000 effector cells was significant (R^2 ^= 0.89, Fig. 8). These data demonstrate that the GrB ELISPOT assay accurately measures the rapid release of GrB by stimulated LAK cells and can enumerate GrB-secreting effector cells.

LAK cells were utilized in the GrB ELISPOT, IFN-γ ELISPOT and ^51^Cr-release assays to evaluate the different assays for measuring innate immunity (Table 2). Both ELISPOT assays were significantly more sensitive than the ^51^Cr-release assay. At effector:target ratios of 20:1-5:1, significant specific lysis can be measured by the ^51^Cr-release assay but the spots per well in the ELISPOT assays were too numerous to count accurately. Significant IFN-γ and GrB secretion was evident at effector:target ratios as low as 0.02:1 (1000 effectors/well) therefore, the optimal ratios for the ELISPOT assays are below the level of sensitivity of the ^51^Cr-release assay (Table 2). However, when the optimal number of LAK cells are used in each individual assay, the amount of GrB and IFN-γ secreting cells in the ELISPOT assays and cytotoxicity in the ^51^Cr-release assay have shown cross-correlation with R^2^ values greater than 0.86 for all combinations. When comparing the two ELISPOT assays, the GrB ELISPOT is more rapid and may be a more direct measure of cytotoxic activity than the IFN-γ ELISPOT. Therefore, the GrB ELISPOT assay can elucidate the frequency of GrB secreting cells in primary LAK cultures and can be applied to measure innate immunity in clinically relevant samples.

**Table 2:** Comparison of the GrB ELISPOT, IFN-γ ELISPOT and ^51^Cr-release assays for measuring Lymphokine Activated Killer (LAK) cell activity.

Effector:Target Ratio	^51^Cr-Release Assay^a ^(% Cytotoxicity ± SE)	IFN-γ ELISPOT Assay^b ^(Spots/well ± SE)	GrB ELISPOT Assay^c ^(Spots/well ± SE)
20:1	42 ± 10	TNTC^d^	TNTC
10:1	30 ± 10	TNTC	TNTC
5:1	22 ± 6	TNTC	TNTC
2.5:1	10 ± 3	TNTC	TNTC
1:1	5 ± 1	TNTC	TNTC
0.4:1	4 ± 1	227 ± 16	282 ± 18
0.2:1	2 ± 1	182 ± 8	193 ± 12
0.1:1	2 ± 2	121 ± 14	117 ± 12
0.04:1	1 ± 0	61 ± 13	54 ± 11
0.02:1	1 ± 0	29 ± 8	37± 7

^a ^The ^51^Cr-release assay was performed with the effector:target cell ratio as specified for 4 h at 37°C. Data presented as average cytotoxicity ± SE (n = 3).

^b ^The IFN-γ ELISPOT assay was performed with K562 cells at a constant number of 5 × 10^4 ^cells per well and effector:target cell ratio as specified for 24 h at 37°C. Data presented as average spots/well ± SE (n = 3).

^c^The granzyme B ELISPOT assay was performed with K562 cells at a constant number of 5 × 10^4 ^cells per well and effector:target cell ratio as specified for 4 h at 37°C. Data presented as average spots/well ± SE (n = 3).

^d^TNTC= Too numerous to count accurately.

## Discussion

NK cells are a key part of innate immunity due to their ability to secrete cytokines and mediate cytolytic activity and act as an important first line of defense against virally infected cells and tumor cells [30]. Although cytokine secretion by NK cells plays a role in regulating the adaptive immune response, cell-mediated cytotoxicity is the major effector function of NK cells. NK cells can mediate cytotoxicity by two main pathways, FasL-mediated and granule-mediated. The granule-mediated pathway, however, is dominant in NK cells with the release of GrB as one of the key factors in the lethal interaction that kills virus-infected and tumor cells [2,5,7,8,10,15-19]. Therefore, evaluating the secretion of this molecule provides an indirect measure of cell-mediated cytotoxicity mediated via the release of granules.

The GrB ELISPOT assay has been previously applied to enumerate the frequency of antigen-specific T cells and this release of GrB by T cells was indicative of cytolytic ability [24,25,31]. Moreover, the GrB ELISPOT assay requires significantly less effector cells than the standard ^51^Cr-release assay [24,25]. In this study, we demonstrated that the GrB ELISPOT assay can be utilized to determine the frequency and potential cytolytic ability of innate immune cells. When TALL-104 or LAK cells were used as effectors, our results were in excellent agreement with results obtained when GrB-transfected CHO cells, T-cell lines or CTL stimulated *in vitro*, were employed as effector cells [24,25]. TALL-104 or LAK cells secreted significant amounts of GrB only when stimulated with an NK-sensitive target cell line indicating that the GrB ELISPOT assay primarily detected stimulated, and not spontaneous (constitutive), release of GrB. Although assessing GrB release is an indirect measure of target cell lysis, a similar trend between the number of GrB spots per well in the ELISPOT assay and specific lysis in the ^51^Cr-release was observed. Therefore, the GrB ELISPOT assay is a viable alternative to the ^51^Cr-release assay for measuring MHC non-restricted cytolytic activity.

Preformed granules that contain mature GrB are constitutively expressed in NK cells, thus NK cells are always armed with functional GrB. Preformed GrB is rapidly released upon recognition and conjunction of the effector cell and this process is Ca^2+^-dependent [18,19,32]. In contrast, IFN-γ is produced *de novo *upon activation and is secreted within hours [33]. The differences observed in the pattern of GrB and IFN-γ secretion suggest that the GrB measured in the ELISPOT assay is due to degranulation of pre-formed GrB whereas IFN-γ secretion results from protein synthesis *de novo*.

To confirm that GrB measured in the ELISPOT assay was released via degranulation, effector cells were treated with inhibitors of cellular secretion: brefeldin A, BAPTA-AM and EGTA. Brefeldin A reversibly disrupts protein translocation from the endoplasmic reticulum to the Golgi apparatus, blocking the production and subsequent secretion of newly synthesized proteins [34]. BAPTA-AM and EGTA are both Ca^2+^-chelating agents and therefore can block Ca^2+^-dependent degranulation. Cell permeant BAPTA-AM contains acetoxymethyl ester groups that are removed by cytosolic nonspecific esterases, trapping the chelator within the cell. In a series of experiments, treatment of effector cells with brefeldin A significantly decreased IFN-γ, but not GrB secretion, whereas, BAPTA-AM abrogated GrB secretion but its effect on IFN-γ secretion was not apparent (data not shown). EGTA, which sequesters extracellular Ca^2+^, could also abrogate GrB secretion measured in the ELISPOT assay. However, significantly higher concentrations of EGTA (4–40 mM) than BAPTA-AM were needed to block GrB secretion (data not shown). These data are in good agreement with recently published findings [35] which demonstrate that higher concentrations of EGTA are needed to block degranulation compared to BAPTA-AM. Although adherence of cytolytic cells to their targets is a prerequisite for granule release, this interaction is dependent on Mg^2+^, but not Ca^2+ ^[19]. Thus, the decrease in GrB secretion after BAPTA-AM and EGTA treatment cannot be attributed to inhibition of adhesion between the effector and target cells, but results from inhibition of degranulation.

Recently, a flow cytometric assay based on CD107a (lysosomal-associated membrane protein-1) mobilization was developed to measure degranulation of cytolytic cells [28,29]. CD107a is a vesicle membrane protein of cytolytic granules that is transiently expressed on the surface of effector cells during degranulation. Correlations between direct lytic ability and surface expression of CD107a on effector cells has been shown, indicating that CD107a expression is a reliable measure of cytolytic capacity [28,29]. In our study, CD107a expression correlated with GrB secretion (R^2 ^= 0.89). Both the release of GrB and the expression of CD107a was observed as early as 30 min after LAK cells were stimulated with relevant target cells, and increased with extended effector-target cell interaction. These data confirm that the GrB ELISPOT assay is an excellent measure of cytotoxic capacity mediated by effector cell degranulation.

When the ELISPOT assays were directly compared to the ^51^Cr-release assay, they demonstrated higher sensitivity with both TALL-104 and LAK cells as effector cells, data consistent with previous studies [24,25,36,37]. The ELISPOT assays enumerate antigen specific lymphocyte frequency by measuring secretion of specific immune proteins engaged in the specific pathway utilized to mediate lysis of target cells, whereas the ^51^Cr-release assay measures cytotoxic ability regardless of the mechanisms of the killing. Therefore, unlike the ^51^Cr-release assay, the ELISPOT assays are both qualitative and quantitative.

It is important to emphasize that ^51^Cr-release and the GrB ELISPOT assay measure different aspects of cell-mediated killing – target cell death and effector cell function, respectively. A limitation of the GrB ELISPOT assay is that it measures degranulation, not direct target cell lysis. As such, degranulation may not always equate to cell death if target cells contain serpin proteinase inhibitor 9 (PI9), a protein that inhibits the proteolytic activity of GrB, [38] or if effector cells are perforin deficient. The GrB ELISPOT assay also does not account for cytotoxicity mediated by FasL pathway. Therefore, when appropriate, the two assays should be used in concert. However, the high sensitivity and specificity of the ELISPOT assay are beneficial for monitoring clinical trials where frequently there are limited numbers of patients' cells available or target cells cannot be effectively labeled. Target cells that resist or do not tolerate labeling, such as some primary tumor cells, can still be utilized in the GrB ELISPOT assay. Additionally, compared to the ^51^Cr-release assay, the GrB ELISPOT provides the precusory frequency of cells with the potential to kill targets. Although a number of flow cytometric assays have been developed to assess target cell cytotoxicity as well as identify the phenotype of the effector cells mediating the immune response, these assays are not as sensitive as the ELISPOT assay.

The use of the IFN-γ ELISPOT assay as a surrogate measure for CTL and NK responses has recently gained increased application as an alternative to the ^51^Cr-release assay [36,39-43]. However, the IFN-γ ELISPOT assay may not be an accurate measure of cytotoxic lymphocytes since 1) non-cytotoxic cells can secrete IFN-γ and 2) CTL with lytic activity do not always secrete IFN-γ [44-48]. The release of GrB is a more specific measure of cytotoxic lymphocytes than IFN-γ because the expression of GrB is restricted to CTL and NK cells [8,49,50]. Therefore, the GrB ELISPOT assay may be a more direct measure of innate immunity compared to the IFN-γ ELISPOT. The high sensitivity and specificity of the ELISPOT assays are beneficial for monitoring clinical trials where frequently there are limited numbers of patients' cells available. As such, simultaneous use of the IFN-γ and GrB ELISPOT assays may provide important immunological insight into patient responses that may then be directly assessed against clinical outcome.

## Conclusion

Our data demonstrate that the GrB ELISPOT assay is a viable alternative to the standard ^51^Cr-release assay to measure granule-mediated cytotoxicity and could be easily adapted to reliably and accurately measure MHC non-restricted cytotoxicity indicative of NK and LAK activity. The GrB ELISPOT assay measured GrB release due to degranulation of stimulated effector cells. Additionally, GrB is a more specific candidate marker than IFN-γ to measure the cytotoxic capacity of innate immune effector cells such as NK cells.

## Competing interests

None declared.

## Author's contributions

KSW and AM designed the study, analyzed the data and drafted the manuscript. KSW carried and prepared the cell lines and performed the majority of the immunogical assays. MWB performed and analyzed the flow cytometric CD107a assay data. SS participated in the design of the study and preparation of the manuscript. TS, DK and MB participated in the design of the study and served as expert advisors. All authors read and approved the final manuscript.
